# Proposed guidelines for appropriate utilization of superficial radiation therapy in management of skin cancers. Zemtsov‐Cognetta criteria

**DOI:** 10.1111/srt.13311

**Published:** 2023-03-30

**Authors:** Alexander Zemtsov, Armand Cognetta, John Marvel, Ann Logan

**Affiliations:** ^1^ University Dermatology Center Muncie Indiana USA; ^2^ Indiana University School of Medicine Indianapolis Indiana USA; ^3^ Florida State University School of Medicine Tallahassee Florida USA; ^4^ Cancer Care Group Indianapolis Indiana USA

**Keywords:** appropriate use criteria, dermatology, radiation, skin cancer

## Abstract

**Objective:**

To develop appropriate use criteria (AUC) for the treatment of basal cell and squamous cell carcinoma by superficial radiation therapy (SRT) technique.

**Material and Methods:**

Delphi‐type discussion of the experts.

**Results:**

Presented in Figure 1.

**Conclusion:**

These AUCs are in compliance both with the position statement of the American Academy of Dermatology (AAD) and the ASTRO Clinical Practice Guideline on this subject. It is further recommended that SRT will be only performed by either a dermatologist who is board certified in Mohs surgery (MDS) and who had adequate SRT training or by radiation oncologists. Hopefully, this publication will stimulate further discussion on this topic.

## 1 INTRODUCTION

The incidence of three major forms of skin cancers, namely basal cell carcinoma (BCC), squamous cell carcinoma (SCC), and melanoma, and associated overall mortality and morbidity continues to increase both in the USA and worldwide.^1^, ^2^, ^3^ These proposed guidelines will primarily focus on our recommendations for appropriate treatment of BCC and SCC by SRT technique. The authors completely agree with the American Academy of Dermatology (AAD) position statement^4^ that surgery should always be considered both the preferred and the first choice treatment option offered to the absolute majority of patients with BCC and SCC. However, some patients, as discussed below, are very poor surgical candidates because of advanced age, being frail and medically unstable, having dementia, or simply because they are categorically refused to have surgery. In these instances, SRT as a second‐choice alternative should be considered. SRT has been used by dermatologists for the treatment of non‐melanoma skin cancers [NMSC] at least since 1902.^5^ The Appropriate Use Criteria (AUC) for the treatment of NMSC by Mohs versus other treatment modalities have been published^6^ and are even available as a smartphone app (its Apple icon is Mohs AUC). On the other hand, even though SRT is now widely used in the US for the treatment of NMSC the criteria for the appropriate use of this technique in the management of BCC or SCC have never been published. In 2019 Nestor and coauthors published “Consensus Guidelines” on the use of SRT for the treatment of NMSC.^7^ Even though we applaud Nestor and coworkers’ efforts their “consensus guidelines”

1. Had no input from radiation oncologists.
2. Did not solicit suggestions from either AAD or Mohs Societies even though its conclusion statement that “superficial radiation therapy should be the first option for treating appropriate types of NMSC tumors in suitable patients”^7^ needs to be reconciled with the AAD position statement mentioned above.
3. Furthermore, their publication did cover too many topics namely, patient safety, radiation dermatitis, dose fractionation, keloid therapy, and even discussion of all forms of radiation available for the treatment of NMSC.^7^

We also reviewed ASTRO guidelines for definite and postoperative radiation therapy of cutaneous SCC and BCC.^8^ These guidelines are excellent in describing indications for postoperative and regional lymph node therapy, dose fractionation schedules, and the use of chemotherapy. However, AUC similar to Mohs AUC was not part of the discussion. This report will laser focus on SRT AUC. Hopefully, this publication will stimulate further dialogue on this topic.

## 2 MATERIAL AND METHODS

Delphi‐type discussion of the experts. Alexander Zemtsov is a board‐certified Mohs surgeon who has been using SRT since 1990 (including in the past also using of ultrasoft Grenz X‐rays); he is also one of the first physicians in the US to develop expertise in high‐frequency skin ultrasonography (HFUS).^9^ Armand Cognetta is a fellowship‐trained Mohs surgeon who is considered the expert in the field of SRT and the main author of the most authoritative book on this subject.^10^ John Marvel and Ann Logan are board‐certified radiation oncologists who in the past few years have specialized and primarily focused on treating NMSC with SRT. We appreciate ASTRO members reviewing this report and their comments were incorporated into our final recommendations.

## 3 DISCUSSION

**FIGURE 1:**
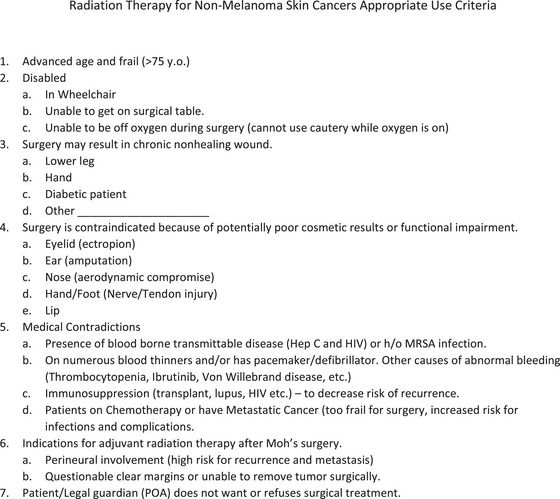
Radiation therapy for non‐melanoma skin cancers appropriate use criteria.

When evaluating patients for SRT we use the criteria shown in Figure 1. Obviously, if a patient categorically refuses surgery SRT is offered as an alternative; on the other hand, as a rule, we do not offer SRT to patients younger than 60 years old, with recurrent tumors, Marjolin ulcers, previously irradiated tumors, and other rare instances that are beyond the scope of this paper discussion (such as basal cell nevus syndrome, etc.). Furthermore, we believe SRT is an excellent option for frail, medically unstable patients (including uncooperative patients with some dementia). SRT is also a good treatment option for patients with previously untreated and very large or multiple untreated skin cancers in the same anatomically important location (eyelid, ear, nose, or lip; on the nose SRT produces remarkably excellent cosmetic results). Finally, as a part of the informed consent discussion, SRT should be presented as a reasonable alternative to surgery when there is a substantial risk of surgical complications (medial canthus or external nasal valve areas and areas where there is a high‐risk injury to tendons/nerves). For each patient referred for SRT, we fill out a form shown in Figure 1 justifying the need for SRT. This form is scanned into the chart and functions as our SRT AUC.

Our final recommendations (not related to SRT AUC criteria).

1. Optimally SRT should be administered only by either a board‐certified Mohs surgeon who had adequate SRT training or by radiation oncologists. Furthermore, appropriately trained physicians should work with medical physicists trained and experienced in SRT. SRT should be offered as a treatment option because of medical indications not because a physician lacks appropriate surgical skills. SRT training may be incorporated into both a dermatology residency curriculum as well as Mohs training. HFUS technique, originally developed by European dermatologists, is used for determining the tumor depth prior to initiation of radiation therapy and for monitoring cancer response to SRT,^11^ is of value to all physicians using SRT.
2. Further studies are needed to document the efficacy of SRT in the management of large, difficult‐to‐resect lentigo maligna/melanoma in situ tumors and the utility of SRT as a postoperative adjuvant therapy to minimize the chance of recurrence in these cancers. The optimal KeV for lentigo maligna is in the 10–20 range – this SRT regimen for this indication is widely used in Europe.

In summary, the authors provided their suggested guidelines for the appropriate use of SRT in the management of skin cancers. Hopefully, this manuscript will stimulate further research and discussion on this subject.

## Data Availability

Research data are not shared.
